# Neonatal jaundice is associated with increased risks of congenital anomalies of the kidney and urinary tract and concomitant urinary tract infection

**DOI:** 10.1038/s41598-024-59943-2

**Published:** 2024-04-25

**Authors:** Hsin-Hsu Chou, Lin-Chih Huang, Shang-Po Shen, Ming-Luen Tsai, Yu-Chia Chang, Hung-Chi Lin

**Affiliations:** 1 Department of Pediatrics, Ditmanson Medical Foundation Chia-Yi Christian Hospital, Chiayi, Taiwan; 2 Department of Bioinformatics and Medical Engineering, Asia University, Taichung, Taiwan; 3 Division of Neonatology, China Medical University Children’s Hospital, China Medical University, No. 2 Yuh Der Road, Taichung, 404 Taiwan; 4 Department of Healthcare Administration, College of Medical and Health Science, Asia University, No. 500, Lioufeng Road., Wufeng, Taichung, 41354 Taiwan; 5 Department of Long-Term Care, College of Health and Nursing, National Quemoy University, Kinmen County, Taiwan; 6 School of Chinese Medicine, College of Chinese Medicine, China Medical University, Taichung, Taiwan; 7 Asia University Hospital, Asia University, Taichung, Taiwan

**Keywords:** Medical research, Risk factors, Urology

## Abstract

The link between neonatal jaundice and urinary tract infection (UTI) remains debated, with congenital kidney and urinary tract anomalies (CAKUT) potentially playing a role. This population-based study aimed to analyze the correlations between neonatal jaundice, CAKUT, and concomitant UTI. The study cohort consisted of 2,078,122 live births from 2004 to 2014. We linked several population-based datasets in Taiwan to identify infants with unexplained neonatal jaundice and their mothers. The primary outcome was the rate of CAKUT occurring within 3 years after delivery, and the presence of concomitant UTI during neonatal jaundice hospitalization. Infants with neonatal jaundice had a significantly higher risk of CAKUT (adjusted odds ratio [aOR] 1.24, 95% confidence interval [CI] 1.11–1.39) during early childhood. Among the subtypes of CAKUT, obstructive uropathy, vesicoureteral reflux and other CAKUT were associated with an increased risk of neonatal jaundice. Infants who underwent intensive phototherapy, had a late diagnosis (> 14 days of postnatal age) or underwent a prolonged duration of phototherapy (> 3 days) exhibited a higher risk of concomitant UTI compared to other infants with jaundice. Our findings indicate a notable association between neonatal jaundice and increased risks of UTIs in the context of CAKUT. This study underscore the importance of vigilant monitoring and timely interventions for neonates presenting with jaundice, while acknowledging the complexity and variability in the progression of CAKUT and its potential connection to UTIs.

## Introduction

The association between neonatal jaundice and urinary tract infection (UTI) has been reported in previous studies^1–6^. Jaundice, especially prolonged jaundice or unexplained jaundice, as an early warning sign of UTI in infancy, has been proposed. A positive urine culture, with sampling by either catheterization or clean catch, has been reported in 3.6–18% of jaundiced infants^1–3,7,8^. Additionally, increased bilirubin levels have been associated with renal cortical damage in jaundiced neonates with UTI^9^. However, jaundice is often observed in the newborn period, with approximately 60–80% of newborns manifesting with clinical jaundice, especially East Asian infants^10,11^. Whether bacterial growth in urine culture in asymptomatic jaundiced infants is coincidence or a consequence remains debated^12–14^. Thus, the performance of routine urine screening for UTI in jaundiced infants is still controversial.

Congenital anomalies in the kidney and urinary tract (CAKUT) is recognized as an important risk factor for UTI, with a prevalence of 25%-55% in infants with UTI^15^. Previous studies have shown that CAKUT accounts for 50%-60% of chronic kidney disease (CKD) and 20–30% of end-stage kidney disease (ESKD) in pediatric patients^16–19^. Among infants with CAKUT, 61% had a UTI before the age of 3 months. In addition, vesicoureteral reflux (VUR) accounted for nearly 60%, and obstructive uropathy accounted for one-third of all CAKUT cases^20^. Recurrent UTI due to CAKUT may predispose children to renal scarring, hypertension and CKD^21,22^. It is important to recognize these patients early to avoid kidney damage, minimize deterioration of renal function and prevent complications of ESKD.

Given the controversy regarding urine screening for UTI in jaundiced infants, there is an urgent need to elucidate the correlation between UTI or underlying CAKUT and neonatal jaundice. We hypothesized that infants with underlying CAKUT may be predisposed to UTI, leading to a higher prevalence of neonatal jaundice. These vulnerable infants remain susceptible to UTI even after the neonatal period and frequently require medical attention due to typical symptoms/signs of UTI, such as fever, dysuria, urination frequency, irritable crying or an abnormal urine odor. Therefore, the aims of this study were (1) to identify the correlation between the prevalence of CAKUT and neonatal jaundice and (2) to identify specific high-risk groups of neonatal jaundice that have an association with concomitant UTI.

## Results

### Demographics of the study cohort

**Figure 1:**
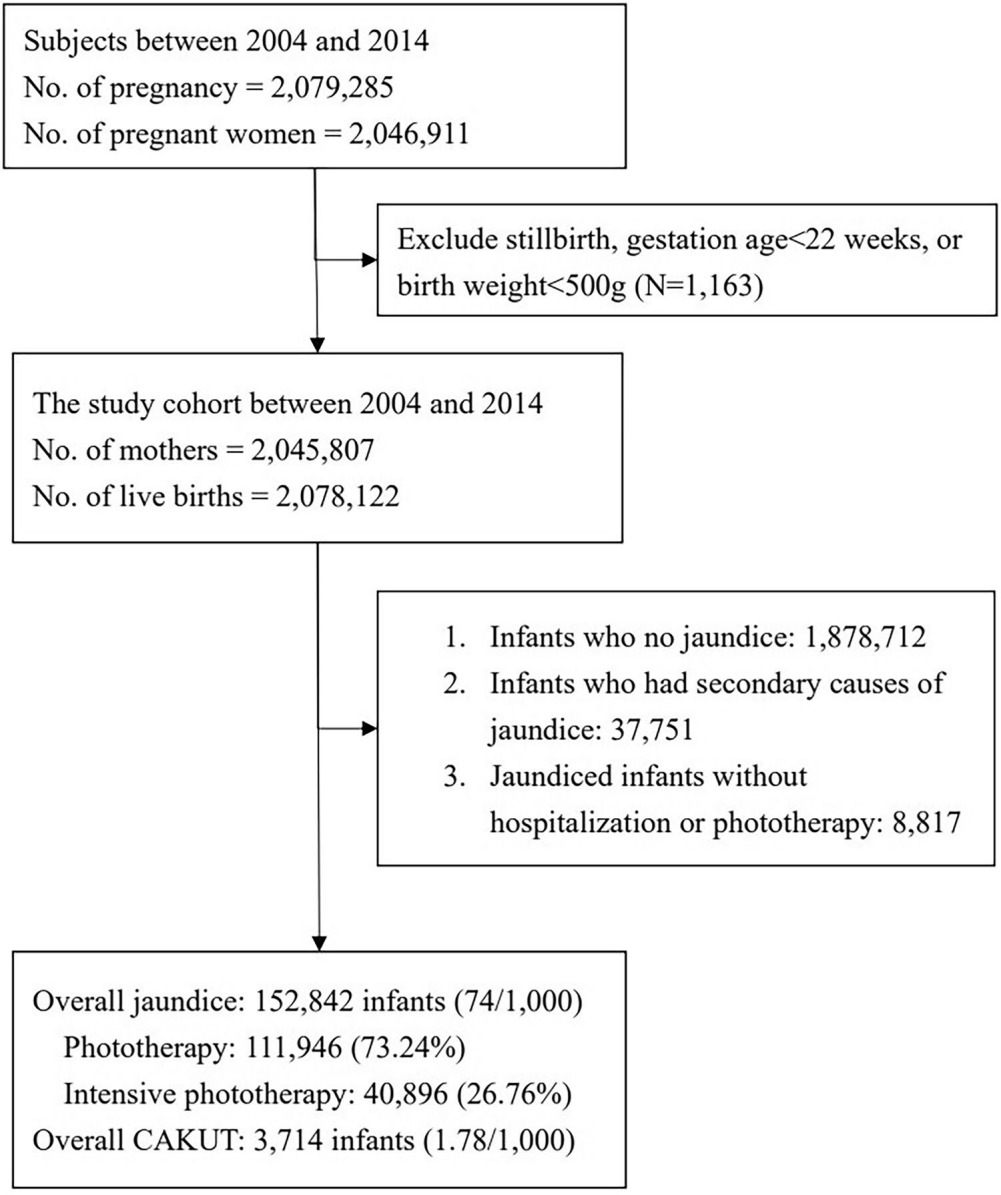
Flow diagram of study cohort selection.

We identified 2,078,122 live birth records from 2,045,807 mothers between 2004 to 2014 after excluding stillbirths, infants born before 22 weeks of gestational age, and those with birth weights of less than 500 g. In total, the study cohort included 152,842 (7.4%) infants who were diagnosed with neonatal jaundice within 8 weeks after birth (Fig. 1). Of these, 111,946 (73.2%) infants underwent regular phototherapy, while 40,896 (26.8%) infants underwent intensive phototherapy and/or blood exchange transfusion.

Table 1 describes the comparison of demographic data between infants with and without neonatal jaundice. Older maternal age (> 30 years), a higher family income, a later birth year (> 2010) and urban residency were more prevalent in the neonatal jaundice group than in the nonjaundice group. The neonatal jaundice group also had higher proportions of males and premature births. The percentages of infants with acute kidney injury, CKD, and those who underwent renal sonography were similar between the jaundiced and non-jaundiced groups. In the non-jaundiced infant group, a relatively low rate (119,945 patients, 6.38%) underwent UTI evaluation via urinalysis or urine culture. Of these, 8,638 (7.2%) infants were diagnosed with UTI during hospitalization. The prevalence rates of UTI in both jaundiced and non-jaundiced infants, with and without CAKUT, were presented in Supplement Table 1.

**Table 1:** Demographic data of infants with and without jaundice.

Variables	Neonatal jaundice		No jaundice		p value
	n	(%)	n	(%)	
Total	152,842	100	1,878,712	100	
CAKUT					< 0.001
No	152,493	99.77	1,875,347	99.82	
Yes	349	0.23	3365	0.18	
Maternal age, years (mean ± SD)	30.80 ± 4.79		30.17 ± 4.82		< 0.001
< 20	2621	1.71	36,923	1.97	
20–24	14,936	9.77	232,879	12.4	
25–29	46,397	30.36	622,620	33.14	
30–34	60,241	39.41	692,410	36.86	
35–39	24,761	16.20	257,363	13.7	
≥ 40	3886	2.54	36,517	1.94	
Birth order					< 0.001
1	108,618	71.07	1,304,485	69.44	
2	39,298	25.71	510,041	27.15	
3 +	4926	3.22	64,186	3.42	
Family income					< 0.001
Low	36,322	23.76	556,045	29.6	
Middle	74,016	48.43	902,995	48.06	
High	42,504	27.81	419,672	22.34	
Offspring gestational age, weeks (mean ± SD)	38.22 ± 1.39		38.35 ± 1.61		< 0.001
22–36	12,182	7.97	145,464	7.74	
37–41	140,525	91.94	1,730,434	92.11	
≥ 42	135	0.09	2814	0.15	
Infant sex					< 0.001
Male	86,082	56.32	967,948	51.52	
Female	66,760	43.68	910,764	48.48	
Year of birth					< 0.001
2004–2006	34,422	22.52	536,411	28.55	
2007–2009	43,970	28.77	510,389	27.17	
2010–2012	43,804	28.66	482,024	25.66	
2013–2014	30,646	20.05	349,888	18.62	
Urbanization level					< 0.001
Urban	104,880	68.62	1,216,644	64.76	
Suburban	39,194	25.64	549,255	29.24	
Rural	8768	5.74	112,813	6.00	
Infants with AKI	57	0.04	672	0.04	0.762
Infants with CKD	28	0.02	314	0.02	0.642
Underwent renal sonography	13,787	9.02	152,594	8.12	0.277

### Associations of CAKUT with neonatal jaundice

The prevalence rates and ORs for CAKUT in infants overall and by subgroups of neonatal jaundice are shown in Table 2. Infants with neonatal jaundice were associated with a higher risk of CAKUT after adjustment for relevant confounders (aOR 1.24, 95% CI 1.11–1.39 for overall jaundice; aOR 1.29, 95% CI 1.13–1.46 for jaundice with regular phototherapy; aOR 1.11, 95% CI 0.89–1.38 for jaundice with intensive phototherapy). Moreover, infants who experienced the onset of neonatal jaundice less than 14 days after delivery were found to have an elevated risk of CAKUT compared to those without jaundice (aOR 1.32, 95% CI 1.15–1.52 for onset of jaundice within 7 days of postnatal age; aOR 1.34, 95% CI 1.09–1.64 for onset of jaundice between 8–14 days of postnatal age; aOR 0.85, 95% CI 0.63–1.15 for onset of jaundice after 14 days of postnatal age). Furthermore, infants who received phototherapy for more than 3 days were also associated a higher risk of CAKUT compared to those without jaundice (aOR 1.09, 95% CI 0.95–1.25 for phototherapy lasting less than 4 days; aOR 1.58, 95% CI 1.29–1.93 for phototherapy lasting 4–6 days; aOR 2.31, 95% CI 1.50–3.55 for phototherapy lasting more than 6 days).

**Table 2:** Prevalence rates and odds ratios for CAKUT overall and in subgroups of neonatal jaundice, 2004–2014.

	CAKUT	Prevalence (95% CI), per 1000 live births	Odds ratio (95% CI)	
	*n*		Unadjusted	Adjusted^a^
No jaundice	3365	1.8 (1.7–1.9)	Reference	Reference
Overall jaundice	349	2.3 (2.1–2.5)	1.28 (1.14–1.43)	1.24 (1.11–1.39)
Severity of jaundice				
Regular phototherapy	266	2.4 (2.1–2.7)	1.33 (1.17–1.51)	1.29 (1.13–1.46)
Intensive phototherapy	83	2.0 (1.6–2.5)	1.13 (0.91–1.41)	1.11 (0.89–1.38)
Onset of jaundice				
≦ 7 days	208	2.5 (2.1–2.8)	1.37 (1.19–1.58)	1.32 (1.15–1.52)
8–14 days	97	2.4 (2.0–3.0)	1.36 (1.11–1.67)	1.34 (1.09–1.64)
> 14 days	44	1.5 (1.1–2.1)	0.87 (0.64–1.16)	0.85 (0.63–1.15)
Duration of phototherapy				
≦ 3 days	228	2.0 (1.8–2.3)	1.13 (0.99–1.29)	1.09 (0.95–1.25)
4–6 days	100	2.9 (2.3–3.5)	1.60 (1.31–1.96)	1.58 (1.29–1.93)
> 6 days	21	4.2 (2.6–6.5)	2.37 (1.54–3.65)	2.31 (1.50–3.55)

^a^Adjusted ORs for CAKUT were calculated using a logistic regression model with a generalized estimating equation and all of the variables listed in Table 1 except CAKUT.

### Associations of subtypes of CAKUT with neonatal jaundice

**Figure 2:**
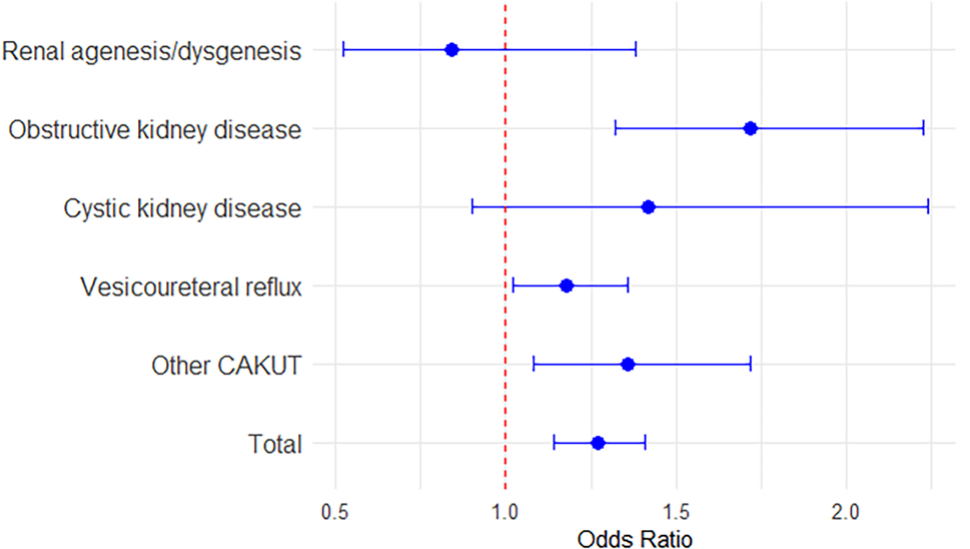
Adjusted odds ratios for subtypes of CAKUT in relation to neonatal jaundice. ORs for CAKUT were adjusted for all of the variables listed in Table 1 plus maternal diabetes.

The association between various subtypes of CAKUT and neonatal jaundice are shown in Fig. 2. Among the five subtypes of CAKUT, infants with neonatal jaundice were found to have increased risks of developing obstructive kidney disease, vesicoureteral reflux and other CAKUT after adjusting for potential confounding factors (aOR 1.65, 95% CI 1.25–2.17 for obstructive kidney disease; aOR 1.17, 95% CI 1.01–1.36 for vesicoureteral reflux; aOR 1.31, 95% CI 1.03–1.67 for other CAKUT). No significant correlation was observed between renal agenesis/dysgenesis or cystic kidney disease and neonatal jaundice.

### Associations of concomitant UTI in relation to maternal and infant characteristics

Of the infants with neonatal jaundice, 68,272 (44.7%) underwent evaluation for UTI through either urinalysis or urine culture. A total of 5024 (3.3%) infants had concomitant UTI during their hospitalization for neonatal jaundice. The prevalence rates and ORs for concomitant UTI in infants in relation to neonatal jaundice are presented in Table 3. Jaundiced infants who received intensive phototherapy exhibited a higher risk of concomitant UTI compared to those who received regular phototherapy (aOR 1.32, 95% CI 1.24–1.40). Jaundiced infants with a late diagnosis of jaundice after 7 days of postnatal age showed an elevated risk of concomitant UTI compared to those diagnosed within 7 days of postnatal age (aOR 1.07, 95% CI 0.99–1.14 for diagnosis of jaundice between 8–14 days of postnatal age; aOR 1.19, 95% CI 1.10–1.28 for diagnosis of jaundice after 14 days of postnatal age). Furthermore, infants who received phototherapy for more than 3 days were found to have a significantly higher risk of concomitant UTI than those for whom phototherapy lasted 3 days or fewer (aOR 1.36, 95% CI 1.28–1.45 for a duration of phototherapy between 4–6 days; aOR 1.44, 95% CI 1.26–1.64 for a duration of phototherapy more than 6 days).

**Table 3:** Prevalence rates and odds ratios for concomitant UTI in subgroups of neonatal jaundice, 2004–2014.

	Concomitant UTI, *n*	Prevalence (95% CI), per 1000 live births	Odds ratio (95% CI)	
			Unadjusted	Adjusted^a^
Severity of jaundice				
Regular phototherapy	3264	67.5 (65.3–69.8)	Reference	Reference
Intensive phototherapy	1760	88.4 (84.5–92.4)	1.34 (1.26–1.42)	1.32 (1.24–1.40)
Onset of jaundice				
≦ 7 days	2613	70.1 (67.5–2.7)	Reference	Reference
8–14 days	1287	74.3 (70.4–78.3)	1.07 (0.99–1.14)	1.07 (0.99–1.14)
> 14 days	1124	82.3 (77.8–87.1)	1.19 (1.11–1.28)	1.19 (1.10–1.28)
Duration of phototherapy				
≦ 3 days	3078	66.5 (64.3 -68.8)	Reference	Reference
4–6 days	1670	88.3 (84.3–92.4)	1.36 (1.28–1.45)	1.36 (1.28–1.45)
> 6 days	276	89.7 (79.9–100.4)	1.38 (1.22–1.57)	1.44 (1.26–1.64)

^a^Adjusted ORs for UTI were calculated using a logistic regression model with a generalized estimating equation and adjusting for all of the variables listed in Table 1 except CAKUT.

## Discussion

In this nationwide population-based cohort study, children with a history of neonatal jaundice exhibited a 24% higher risk of being diagnosed with CAKUT than those without neonatal jaundice before the age of 3 years after adjustment for potential confounding factors. Infants who received intensive phototherapy, experienced a late diagnosis (> 7 days of postnatal age) or underwent prolonged phototherapy (> 3 days) demonstrated an elevated risk of concomitant UTI in comparison to other infants with jaundice. This proactive approach could aid in early identification and management of CAKUT, a condition that may not be immediately apparent in neonates with jaundice but carries significant long-term health implications. While our study identifies a significant association between neonatal jaundice and increased risks of UTI in the context of CAKUT, it is important to emphasize that not all cases of CAKUT lead to UTIs. This observation underscores the multifactorial nature of UTIs in neonates and the complex interplay of factors contributing to their occurrence. Therefore, while our findings highlight a potential risk group, they should be interpreted with caution, considering the diversity in CAKUT presentations and the varying propensity for UTI development in these cases.

Jaundice is a common problem in infants within the first 2 weeks of life, and it has been debated whether the identification of bacterial growth in urine culture represents a coincidental finding or is a contributing cause of neonatal jaundice^12,23^. Additionally, critics have raised concerns about the lack of a control group comprising newborns without jaundice in previous studies examining the association between UTI and neonatal jaundice^23^. While Shahian et al.^3^. reported a UTI prevalence of 12.5% in neonates with unconjugated hyperbilirubinemia occurring within the first week of life compared to 0% in healthy neonates, most prior studies predominantly compared jaundiced infants with both positive and negative urine cultures^1,2,4–7^. Consequently, drawing a definitive conclusion regarding the comparative incidence of UTIs in jaundiced infants versus those without jaundice becomes inherently challenging without an appropriately matched nonjaundice control group. To further clarify these controversial issues, our study sought to evaluate both CAKUT and concomitant UTI as the outcomes of interest among infants with neonatal jaundice. Our study demonstrated an increased risk of CAKUT, an important predisposing factor of UTI, in jaundiced infants in comparison to non-jaundiced infants and may provide indirect evidence that contributes to elucidating the potential correlation between neonatal jaundice and UTI.

The association between neonatal jaundice and UTI may be influenced by several potential mechanisms. One potential mechanism for the association between neonatal jaundice and UTI is hemolysis caused by E. coli and other gram-negative bacteria. This hemolysis, even when mild, can lead to significant hyperbilirubinemia in neonates due to immature conjugation mechanisms, thereby elevating serum bilirubin levels^24^. Cholestasis due to infection and potentially reduced activity of the enzyme glucuronic transferase might contribute to these elevated bilirubin levels^9^. Additionally, fluid depletion, which might occur in the context of neonatal jaundice, can lead to renal hypoperfusion and increase the risk of acute kidney injury and UTIs^25^. This suggests that dehydration or hypohydration, potentially secondary to jaundice, could be a risk factor for UTIs in neonates. While these studies offer some insights, the exact mechanisms linking neonatal jaundice and UTIs remain to be fully elucidated, warranting further research in this area.

Routine urine screening in jaundiced infants has been debated, leading to variations in practices across different regions. The National Institute for Health and Clinical Excellence (NICE) 2010 guidance on neonatal jaundice recommends the utilization of urine culture for investigation in neonates with prolonged jaundice^26^, whereas the American Academy of Pediatrics (AAP) does not recommend urine testing in jaundiced neonates^27^. Our study identified several high-risk groups of neonatal jaundice, including infants subjected to intensive phototherapy, those with delayed diagnoses, and those undergoing prolonged durations of phototherapy. The implications of these findings may provide essential insights for clinicians to identify potential cases of UTI among infants with neonatal jaundice. Prospective controlled trials are imperative to elucidate which specific high-risk subgroups of infants with neonatal jaundice warrant further investigation for UTI.

The prevalence of concomitant UTI in infants with neonatal jaundice in this study was relatively low, with 3.3% of the entire cohort of jaundiced infants affected (7.6% among jaundiced infants who underwent either urinalysis or urine culture), in comparison with the prevalence reported in previous studies, which ranged from 3.6–21.1%^1–7^. The low urinary screening rate may partially explain the low prevalence because only 44.7% of patients underwent either urinalysis or urine culture for investigation of UTI in our study. Differences in inclusion criteria, durations of jaundice, methods of urine collection, and definitions of UTI may contribute to the highly diverse incidence rates of UTI in jaundiced infants. Notably, Chen et al. reported a 5.5% UTI rate among infants with jaundice aged 8 weeks and younger in a tertiary hospital in Taiwan^4^. Sixty-three percent of the jaundiced infants had received urinalysis and were enrolled in the study cohort. One of the possible reasons for the differences in the results between our study and previous reports may be due to the underdiagnosis of UTI in infants with jaundice because urine testing is not routinely performed in Taiwan in jaundiced infants. This may also help to explain the findings of higher risks of CAKUT in jaundiced infants in our study. Underdetection of UTI in asymptomatic jaundiced infants may contribute to the increased risk of future occurrence of UTI and the identification of underlying CAKUT.

When we further classified CAKUT into 5 subgroups, infants with neonatal jaundice exhibited increased susceptibilities to obstructive kidney disease, reflux nephropathy and other CAKUT. However, this association was not evident for renal agenesis/dysplasia or cystic kidney anomalies. The advancement of fetal ultrasonography, which has revolutionized the early detection and management of conditions like CAKUT, suggests that the recommendation for routine renal sonography evaluation in cases of neonatal jaundice should be approached with caution. This is due to limitations in directly applying our study's findings to contemporary clinical practice. The underlying pathophysiology remains poorly understood. We speculate that UTI could be more prevalent in obstructive kidney disease, likely due to urine stasis, and vesicoureteral reflux, stemming from the regurgitation of urine, compared to other subtypes of CAKUT. On the other hand, most current guidelines for febrile UTI do not recommend VCUG for infants experiencing their first episode of UTI^28^. The role of a history of neonatal jaundice and association with VUR and the optimal timing of VCUG examinations in infants with febrile UTI should be investigated through further studies.

### Strengths and limitations

The major strength of this study was the minimal selection bias due to data linkage among several large nationwide population-based datasets, which allowed for reliable analysis of the correlation of neonatal jaundice with CAKUT and concomitant UTI. These datasets have been considered complete and valid in previous studies^29–32^.

Several limitations should be addressed. First, the follow-up period was confined to the first 3 years of life, which may have potentially induced an underestimation of the occurrence of both CAKUT and UTI. Nevertheless, such underidentification is likely of marginal significance, given that a prior study conducted in Taiwan demonstrated that most CAKUT diagnoses manifested prior to the age of three^16^. Second, we used ICD-9-CM and ICD-10-CM codes for the identification of CAKUT and UTI, which may have introduced misclassification bias due to the absence of comprehensive diagnostic reports, including confirmatory procedures such as renal ultrasonography and VCUG for CAKUT, as well as clinical laboratory data, such as urinalysis or urine culture results, for the diagnosis of UTI. To minimize this misclassification, we adopted stringent inclusion criteria which mandated a minimum of two outpatient visits or one hospital admission featuring ICD-9-CM or ICD-10-CM codes, as in previous studies^30,33^, to circumvent the inclusion of clinically nonsignificant CAKUT or UTI cases. Third, the higher risk of CAKUT within the jaundiced group may be a reflection of detection bias because jaundiced infants could be more likely to undergo diagnostic evaluations, such as renal ultrasonography or VCUG, than their nonjaundiced counterparts due to concomitant UTI. However, due to the relatively low incidence of UTI in our cohort of jaundiced infants, and because the diagnosis of most CAKUT cases occurred after the neonatal period, this detection bias should be trivial. Fourth, jaundice severity was delineated primarily by the application of phototherapy, as comprehensive serum bilirubin level data were not available. We were also unable to analyze the distinction between unconjugated and conjugated jaundice due to data limitations. Finally, there is the potential for residual confounding due to the lack of data regarding certain maternal lifestyle factors, such as smoking and alcohol consumption, although their prevalence rates are comparably low in Taiwan^34^.

## Conclusions

Infants with neonatal jaundice were associated with elevated risks of concomitant UTI during hospitalization and CAKUT in early childhood. Given the susceptibility of infants with CAKUT to recurrent UTIs and their pronounced vulnerability to CKD or even ESKD, screening for CAKUT in these infants may facilitate timely intervention and subsequent monitoring to mitigate the progression of renal function deterioration and prevent or delay complications linked to CKD. While jaundiced infants may be at a heightened risk of UTI and CAKUT, caution should be taken in assuming a direct causal relationship. Future prospective study is necessary to further elucidate the links between neonatal jaundice, CAKUT and UTI.

## Methods

### Data sources

Three nationwide population-based datasets were used for analysis in this study: the Taiwan Maternal and Child Health Database (TMCHD, 2004–2017), the Birth Certificate Application (BCA, 2004–2017) database, and the National Health Insurance Research Database (NHIRD, 2000–2017). The TMCHD contains encrypted personal identification numbers for all live newborns and their parents, which enables interlinkage among the above datasets and therefore provides parent–offspring linkages in Taiwan^35^. The BCA database includes all live births and reported stillbirths reported by medical practitioners in all hospitals or clinics in Taiwan. Medical facilities or midwives must inform the Household Registration Office and Health Promotion Administration of relevant information within 7 days of birth in Taiwan^36^. This database provides information about maternal characteristics (e.g. nationality, age at delivery, risk factors for pregnancy), the perinatal period (e.g. method of delivery, Apgar score and complications of delivery) and newborn characteristics (e.g. sex, gestational age, birth weight, birth order, single/multiple birth, and congenital anomalies). Most information from the databases has been validated, and a high level of information integrity has been reported in previous studies^29–31^. The National Health Insurance (NHI) program has been implemented since 1995 in Taiwan. It is a mandatory and single-payer system that covers more than 99% of the whole population of Taiwan, and over 96% of medical facilities are contracted with the NHI Administration to provide health care services^37,38^. The aforementioned datasets were established and are updated and supervised by the Center for Data Science, Ministry of Health and Welfare, Taiwan^39^. Both inpatient and outpatient medical claims from the NHIRD were used in this study. The privacy of all patients was protected by the encryption of personal information, and anonymous identification numbers associated with relevant claims information were provided to the researchers. This study fulfilled the condition for exemption and was approved by the Institutional Review Board (IRB) of Ditmanson Medical Foundation Chia-Yi Christian Hospital (CYCH-IRB2022024) and all research was performed in accordance with the Declaration of Helsinki. Due to privacy regulations, information on the beneficiaries was anonymized before being released to the researchers; therefore, the IRB of Chia-Yi Christian Hospital approved informed consent was not required.

### Study population

This was a nationwide retrospective cohort study. All live newborns with a gestational age of more than 22 weeks and a birth weight of more than 500 g in Taiwan were enrolled from January 1, 2004, to December 31, 2014. We identified child–parent pairs through exact deterministic linkage to the TMCHD by the encrypted unique identification number of the child^32^. We obtained the details of both maternal and neonatal characteristics from the BCA database and clinical visit data of both the mothers and children from medical claims in the NHIRD using encrypted personal identification numbers.

### Exposure

The main exposure variable in our study was neonatal jaundice with unknown etiology, which was identified using codes from the clinical modification of the International Classification of Diseases, Ninth Revision (ICD-9-CM) and Tenth Revision (ICD-10-CM) from both inpatient and outpatient claims (ICD-9-CM code: 774.6, ICD-10-CM code: P59.9 for neonatal jaundice). Neonates with secondary causes of jaundice, including biliary atresia (ICD-9-CM code: 751.61, ICD-10-CM code: Q44.2), biliary tract disorder (ICD-9-CM code: 576, ICD-10-CM code: K83), viral hepatitis (ICD-9-CM code: 070, ICD-10-CM code B15-18), hemolytic disease of newborn (ICD-9-CM code 773, ICD-10-CM code P55), hypothyroidism (ICD-9-CM code: 243, ICD-10-CM code: E03.0–1), and galactosemia (ICD-9-CM code: 271.1, ICD-10-CM code: E74.21), were excluded from the cohort. To prevent the inclusion of infants with clinically nonsignificant neonatal jaundice, infants were enrolled only if phototherapy (procedure code: 57106C) was performed during hospital admission within 8 weeks after birth. Infants with neonatal jaundice were further classified into 2 subgroups according to whether they received intensive phototherapy (procedure code: 57117B).

### Outcomes

The primary outcome was the diagnosis of any form of CAKUT, which included congenital anomalies of the urinary system and primary VUR. We divided CAKUT into 5 subgroups, including renal agenesis/dysplasia (ICD-9-CM codes: 753.0, 753.15; ICD-10-CM code: Q60), obstructive kidney disease (ICD-9-CM codes: 753.2, 753.6; ICD-10-CM codes: Q62, Q64.2–3), cystic kidney disease (ICD-9-CM code: 753.1 except 753.15; ICD-10-CM code: Q61), reflux nephropathy (ICD-9-CM code: 593.7; ICD-10-CM code: N13.7), and other CAKUT (ICD-9-CM codes: 753.3–9 except 753.6; ICD-10-CM codes: Q63-64 except Q64.2–3). To prevent the overestimation of non-CAKUT cases or the inclusion of cases of clinically nonsignificant forms of CAKUT, we included only those with the diagnosis recorded for at least one hospital admission or at least 2 outpatient clinic visits at least 1 month apart during the follow-up period. Infants with primary VUR were defined as only those who had received voiding cystourethrography (VCUG), and the diagnostic code for VUR (ICD-9-CM 593.7) was identified after VCUG examination. Infants with any secondary form of VUR due to neurogenic bladder (ICD-9-CM code 344.61, ICD-10-CM code G83.4), bladder neck obstruction (ICD-9-CM code 596.0, ICD-10-CM code N32.0), or functional disorder of the urinary bladder (ICD-9-CM code 596.4–5, 599.6, ICD-10-CM codes N13.9, N31.x, N36.44) were also excluded. Since the majority of CAKUT cases were diagnosed before the age of 3 years^30^, we followed each infant for 3 years after birth to ensure that each infant had the same follow-up period to avoid possible information bias.

The secondary outcome of interest was the occurrence of concomitant UTI, defined as an ICD code for first UTI or acute pyelonephritis (APN) (ICD-9-CM codes: 590.x, 595.x, 597.x, 599.0, ICD-10-CM codes N10, N30, N34, N39.0) recorded during the same hospitalization for neonatal jaundice.

### Covariates

Several relevant covariates that were found to be potentially associated with the risk of CAKUT in previous studies^40–44^ were included in our analysis. Those covariates included maternal characteristics (age at delivery, birth order, family income, maternal diabetes) and infant characteristics (gestational age, sex, birth year, urbanization level of residence at the time of birth, CKD, AKI). Maternal age was categorized as < 20, 20–24, 25–29, 30–34, 35–39, and ≥ 40 years. Gestational age was classified as 22–36, 37–41, and ≥ 42 weeks. Maternal diabetes mellitus (DM) was defined as the diagnosis of either pregestational DM (ICD-9-CM code: 250.x, ICD-10-CM codes E10-13) or gestational DM (ICD-9-CM code: 648.0, ICD-10-CM code O24.x) recorded for at least one hospital admission or at least 3 outpatient clinic visits occurring at least 1 month apart. We classified maternal residential locations into urban, suburban, and rural areas according to the urbanization stratification guidelines published by the Taiwan National Health Research Institute^45^. Adjustment for geographic location helped reduce the effects of urban–rural differences on accessibility to medical health services^46^. Adjustment for year of birth reduced the difference between the utilization of diagnostic tools, such as renal sonography or VCUG, and different time trends for CAKUT in Taiwan. In turn, these adjustments helped reduce the effects of ascertainment bias. CAKUT, including VUR and constipation, was also identified as a potential risk factor for UTI in our analysis.

### Statistical analysis

Categorical variables among the characteristics of mothers and offspring with and without jaundice in the cohort are presented as counts and proportions. We compared these variables with chi-square or Fisher’s exact tests as necessary. Continuous variables are presented as mean values and standard deviations (SDs) and were compared between infants with or without jaundice using the independent t test. The prevalence rates of CAKUT and concomitant UTI in infants with and without neonatal jaundice were calculated. Confidence intervals (CIs) were calculated using the Poisson approximation to the binomial distribution. Potential confounding factors for each outcome of interest were adjusted using logistic regression models. The results are reported as adjusted odds ratios (aORs) and 95% CIs for CAKUT and concomitant UTI in offspring. To address possible within-participant correlations due to the inclusion of infants born to the same mother or infants who lived in the same geographic area, a logistic regression model with a generalized estimating equation was used to obtain robust standard error estimates^47^. All statistical analyses were performed with SAS software, version 9.4 (SAS Institute, NC, USA), and statistical significance was defined as a two-tailed p value of less than 0.05.

### Ethical approval

This study fulfilled the condition for exemption and was reviewed and approved by the Institutional Review Board (IRB) of Ditmanson Medical Foundation Chia-Yi Christian Hospital (CYCH-IRB2022024).

### Supplementary Information

Supplementary Table 1.

## Data Availability

Data are available from the Taiwan Maternal and Child Health Database (TMCHD) published by Center for Data Science, Ministry of Health and Welfare, Taiwan. Due to legal restrictions imposed by the government of Taiwan in relation to the “Personal Information Protection Act”, data cannot be made publicly available. Requests for data can be sent as a formal proposal to the TMCHD.
