# Supplementary material for: Neonatal jaundice is associated with increased risks of congenital anomalies of the kidney and urinary tract and concomitant urinary tract infection
Source: Sci Rep. 2024 Apr 25;14:9520. doi: 10.1038/s41598-024-59943-2 (PMC11045864; doi:10.1038/s41598-024-59943-2)
Supplement: Supplementary file 1 — Supplementary Table 1. [file 41598_2024_59943_MOESM1_ESM.docx]

Supplement Table 1. Prevalence rates of UTI in infants with and without jaundice

| Variables | UTI | Total* | | Prevalence rate (95% CI) |
| --- | --- | --- | --- | --- |
|  |  | n | % |  |
| All infants | 13,662 | 188,217 | 0.73 | 72.59 (71.42-73.76) |
| No CAKUT | 13,481 | 186,931 | 0.72 | 72.10 (70.90-73.30) |
| CAKUT | 181 | 1,286 | 0.14 | 140.70 (122.20-161.00) |
| No jaundice | 8,638 | 119,945 | 0.72 | 72.03 (70.57-73.49) |
| No CAKUT | 8,472 | 118,870 | 0.71 | 71.30 (69.80-72.70) |
| CAKUT | 166 | 1,075 | 0.15 | 154.40 (133.30-177.40) |
| Overall jaundice | 5,024 | 68,272 | 0.74 | 73.59 (71.63-75.55) |
| No CAKUT | 5,009 | 68,061 | 0.74 | 73.60 (71.60-75.60) |
| CAKUT | 15 | 211 | 0.71 | 71.10 (40.30-114.50) |

*Indicates the number and percentage of infants who received UTI evaluation with either urinalysis or urine culture.

Abbreviation: CAKUT, congenital anomalies of kidney and urinary tract; UTI, urinary tract infection.
